# Evaluation of prognostic factors and the role of chemotherapy in unfavorable carcinoma of unknown primary site: a 10-year cohort study

**DOI:** 10.1186/1756-0500-5-70

**Published:** 2012-01-26

**Authors:** Kuo-Wei Chen, Chia-Jen Liu, Hsueh-Ju Lu, Cheng-Hwai Tzeng, Jin-Hwang Liu, Tzeon-Jye Chiou, Chueh-Chuan Yen, Wei-Shu Wang, Ta-Chung Chao, Hao-Wei Teng, Ming-Huang Chen, Chun-Yu Liu, Peter MH Chang, Muh-Hwa Yang

**Affiliations:** 1 Division of Hematology and Oncology, Department of Medicine, Taipei Veterans General Hospital, Taipei, Taiwan; 2 Division of Transfusion Medicine, Department of Medicine, Taipei Veterans General Hospital, Taipei, Taiwan; 3 Department of Medicine, National Yang-Ming University Hospital, Yi-Lan, Taiwan; 4 Faculty of Medicine, National Yang-Ming University, Taipei, Taiwan

## Abstract

**Background:**

Carcinoma of unknown primary site (CUP) has a poor prognosis and the prognostic factors in these patients are not well established. Furthermore, there are no selection criteria for patients who should benefit from chemotherapy.

**Methods:**

The medical records of 179 CUP patients who were treated at Taipei Veterans General Hospital from 2000 to 2009 were reviewed. Factors associated with survival were determined by Kaplan-Meier analysis. Differences between the groups with and without palliative chemotherapy were analyzed.

**Results:**

Univariate analysis revealed multiple prognostic factors, including performance status, lung metastasis, number of metastatic organs, serum albumin, corrected serum calcium, lactate dehydrogenase (LDH), sodium, and cholesterol levels, palliative chemotherapy, and white blood cell and lymphocyte counts. Multivariate analysis showed that performance status < 2, serum albumin level ≥ 3.5 g/dl, corrected serum calcium level < 10.7 mg/dl, single metastatic organ, and palliative chemotherapy were independent factors of better prognosis. Patients with better performance status, higher serum albumin, and lower serum LDH levels had significantly greater benefit from palliative chemotherapy.

**Conclusions:**

Certain patients with unfavorable CUP will have better survival. Identification of patients with unfavorable CUP who could benefit from palliative chemotherapy warrants future prospective studies.

## Introduction

Cancer of unknown primary site (CUP) is defined as a histologically proven metastatic malignant tumor whose primary site cannot be identified after thorough pre-treatment work-up [1]. It is the seventh most prevalent cancer in the world and the fourth commonest cause of cancer death in both men and women [2]. CUP accounts for 2.3%-4.2% of cancers in either gender. Previous studies suggest that CUP patients should be categorized into favorable and unfavorable groups before appropriate management is provided [3-5]. In the favorable group, individual treatment according to the possible primary site, such as poorly differentiated carcinoma with midline distribution, papillary adenocarcinoma of the peritoneal cavity in women, adenocarcinoma involving only axillary lymph nodes in women, and squamous cell carcinoma involving cervical lymph nodes, achieves longer survival [6]. However, almost 85% of CUP patients fall into the unfavorable group, in which chemotherapy is controversial [7]. In last 10 years, further categorization with newly identified prognostic factors, such as Eastern Cooperative Oncology Group (ECOG) performance status ≥ 2, more than one organ metastasis, high serum LDH, and low albumin levels, reflects significantly poor survival within the unfavorable CUP category [7-11]. It remains unknown whether or not the unfavorable CUP patient with a good prognostic factors is a suitable candidate for palliative chemotherapy, which may further improve survival. One possible reason for this confusion is that there are a large variety of treatment outcomes defined between cancer center-based and registry-based studies [7,8], making meaningful comparisons and decisions based on the results difficult. Additionally, previous studies of single regimens did not make clear clarification of CUP patients according to the new prognostic factors [11].

One of the benefits of medical transfer system in Taiwan is that most of the suspected CUP patients will be referred to a major medical center before a definitive diagnosis of CUP is made. This phenomenon enables us to provide more comprehensive evaluation and give palliative chemotherapy, radiotherapy, or new treatment strategies directed against tumor under the discretion of the medical oncologists. In this study, we analyzed the prognostic factors including clinical and biochemical variables of patients with unfavorable CUP at a single tertiary medical center. This is the first large retrospective cohort analysis of unfavorable CUP in Asian population. The results of this article might help clinicians to make better individualized therapeutic plans for the treatment of patients with unfavorable CUP.

## Methods

**Figure 1:**
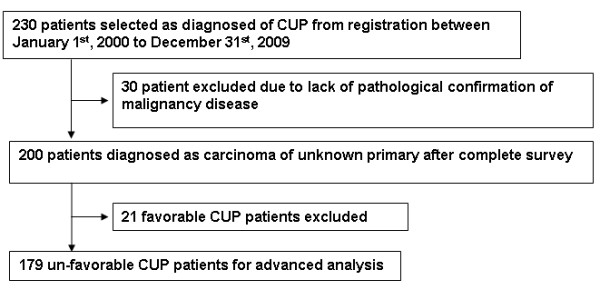
**Patient enrollment**. Patient enrollment scheme for unfavorable CUP (cancer of unknown primary site) study.

We reviewed the medical records of Taipei Veterans General Hospital from January 1^st^, 2000 to December 31^st^, 2009 and identified 230 cases of CUP. Of these, 30 patients were excluded due to lack of pathologic confirmation. Another 21 patients were excluded because they had documented favorable CUP; they included women with lone axillary lymph nodes containing adenocarcinoma, poorly differentiated or undifferentiated carcinoma with characteristics of extragonadal germ cell tumor syndrome, women with diffuse peritoneal carcinomatosis (papillary adenocarcinoma), squamous cell carcinoma involving upper cervical lymph nodes, and squamous cell carcinoma involving solitary inguinal lymph node. Finally, 179 patients with unfavorable CUP were enrolled for advanced analysis of prognostic factors and response to treatment (Figure 1). The ethical approval of this retrospective study had been done by institutional review board (IRB), Taipei Veterans General Hospital with the code number of 2011-03-025IC.

The laboratory parameters were collected within the period of 1 week before and 2 weeks after the definite pathologic diagnosis. The corrected serum calcium levels were calculated as: serum calcium level + 0.8 × (4.0 - patient's albumin level).

The Kaplan-Meier method was used to generate survival curves. Univariate analysis of various parameters for overall survival was done with the Log rank test, and a p < 0.05 (two-tailed) was considered significant. Multivariate Cox regression analyses were performed subsequently using backward, stepwise selection to derive a multivariate model for significant predictors. Overall survival was defined as the time from pathological diagnosis until either death or last follow-up. The independent *t *test and chi squared test were performed to evaluate the characteristic differences between chemotherapy and non-chemotherapy groups, as appropriate. Statistical analyses were done using SPSS software version 17 (SPSS Inc., Chicago, IL) *P *< .05 was considered a statistically significant difference.

## Results

### Patient characteristics

Of the 179 patients studied, 128 were men (71.5%) and 51 were women (28.5%). Their median age was 73 years (range, 30-98 years). The clinical and pathological characteristics of the patients are shown in Table 1. The most common histological diagnoses were unclassifiable carcinoma (83/179, 46.4%) and adenocarcinoma (71/179, 39.7%); thirty-one (17.3%) patients had histologically poorly differentiated or undifferentiated carcinoma. About 52% (93/179) of patients had an ECOG performance status ≥ 2. The most common organs in which tumors were initially diagnosed were bone (26.3%), lymph nodes (24%), and liver (21.2%). After thorough imaging studies, lymph nodes, lungs, liver, and bones were the most frequently involved organs. Approximately one hundred and eighteen patients (66%) received palliative chemotherapy. After a median follow-up of 7.2 months (0.0-64.3 months), the median overall survival was 6.2 months (0.0-64.3 months). The 1-year and 2-year survival rates were 37.2% and 23.8%.

**Table 1:** Characteristics of patients with unfavorable cancer of unknown primary site (n = 179)

Characteristic	No. of patients	%
Median age at diagnosis, year (range, IQR)	73 (30-98, 58-80)	
Gender		
Male	128	71.5
Female	51	28.5
Histology		
Carcinoma, unclassifiable	83	46.4
Adenocarcinoma	71	39.7
Squamous cell carcinoma	6	3.4
Neuroendocrine carcinoma	12	6.7
Others	7	3.9
Histologic grade		
Poorly differentiated or Undifferentiated	31	17.3
Unspecified	148	82.7
Performance status		
0	19	10.6
1	67	37.4
2	44	24.6
3	27	15.1
4	22	12.3
Initial site at diagnosis		
Liver	38	21.2
Lung	23	12.8
Bone	47	26.3
Central nervous system	7	3.9
Peritoneum	18	10.1
Lymph nodes	43	24
Others	3	1.7
Sites of disease involvement		
Liver	80	44.7
Lung and pleural	87	48.6
Bone	74	41.3
Central nervous system	14	7.8
Lymph nodes	107	59.8
No. of metastatic organs		
1	40	22.3
2	52	29.1
> 2	87	48.6
Treatment		
Chemotherapy	118	66.0
Radiotherapy	52	29.1
Tyrosine kinase inhibitors	2	1.1
Chemotherapy regimens		
Cisplatin-based	89	75.4
5-FU-based	51	43.2
Etoposide-based	45	38.1
Gemcitabine-based	17	14.4
Taxane-based	13	11.0
Oxaliplatin-based	11	9.3

IQR, inter-quartile range.

### Prevalence and mortality rate

**Figure 2:**
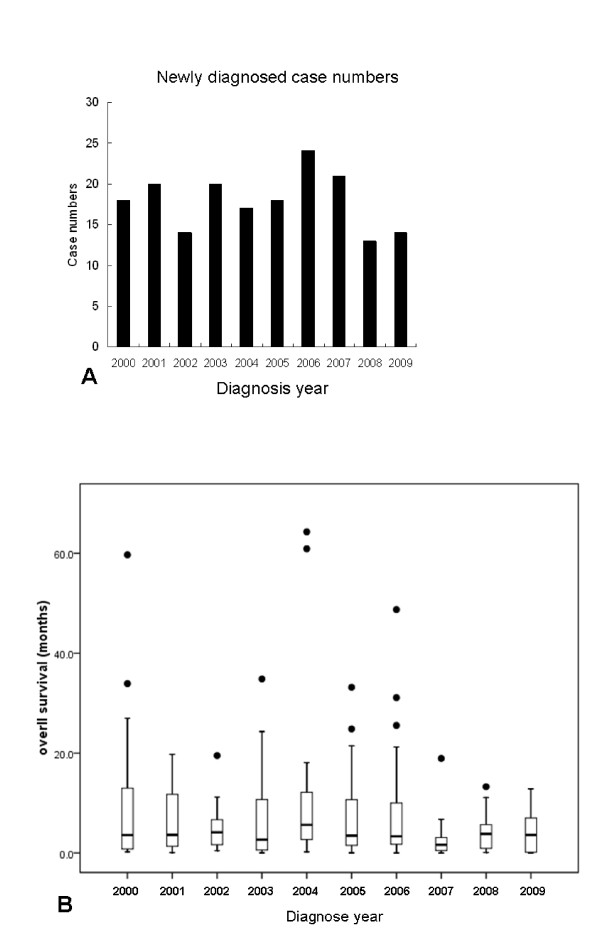
**Case number distribution and survival curve**. (A) Case numbers of patients with unfavorable CUP (cancer of unknown primary site) each year from 2000 to 2009. Thirteen to 24 cases of unfavorable CUP were diagnosed each year. (B) No trend in median overall survival per diagnosis year was observed.

In order to see the differences in occurrence rate and outcome between each year, in Figure 2A, we charted CUP patient numbers diagnosed yearly from 2000 to 2009 as well as median overall survival of patients diagnosed each year in Figure 2B. The case numbers each year were steady and there was no obvious trend of survival improvement or deterioration during the last decade.

### Univariate and multivariate analysis of prognostic factors

The results of univariate analysis are shown in Table 2. The following clinical variables predicted significantly shorter overall survival: ECOG performance status ≥ 2 (*P *< .001), presence of lung metastases (*P *= .005), ≥ 2 metastatic sites (*P *= .028), albumin level < 3.5 g/dl (*P *< .001), corrected calcium level ≥ 10.7 mg/dl (*P *= .006), LDH level ≥ 250 U/L (*P *= .001), serum sodium level < 135 mmol/l (*P *< .001), serum cholesterol level < 150 mg/dl (*P *= .014), and lymphocyte count < 700/μL (*P *= .003).

**Table 2:** Univariate and multivariate analysis of overall survival (OS) of patients with unfavorable cancer of unknown primary site (n = 179)

Variable	N	Median OS, months	Univariate *P *	Multivariate		
				
				HR	95% CI	*P*
Age, years						
< 65	60	5.33	.309			
≥ 65	119	6.47				
Gender						
Male	128	5.63	.467			
Female	51	6.47				
Performance status				2.025	1.294-3.168	.002
0-1	86	13.37	< .001			
2-4	93	3.47				
Body mass index						
< 24	83	5.33	.223			
≥ 24	53	11.8				
Histology						
Adenocarcinoma	71	4.47	.402			
Carcinoma, unspecified	83	6.47				
Squamous cell carcinoma	6	59.7				
Others	19	11.1				
Histologic grade						
Poorly differentiated or Undifferentiated	31	7.03	.869			
Unspecified	148	5.63				
Liver metastases						
Yes	80	5.1	.236			
No	99	7.7				
Lung metastases						
Yes	87	4.43	.005			
No	92	9.2				
Bone metastases						
Yes	74	5.33	.255			
No	105	6.43				
Lymph node metastases						
Yes	107	6.17	.658			
No	72	6.47				
No. of metastatic organs				1.927	1.147-3.237	.013
1	40	18.07	.028			
≥ 2	139	5.33				
Radiotherapy						
Yes	52	11.1	.105			
No	127	5.07				
Chemotherapy				3.211	2.009-5.132	< .001
Yes	118	9.2	< .001			
No	61	1.63				
Albumin				2.216	1.440-3.409	< .001
< 3.5 g/dl	66	2.77	< .001			
≥ 3.5 g/dl	113	11.1				
Estimated creatinine clearance						
< 60 ml/min	75	6.43	.806			
≥ 60 ml/min	74	6.17				
Corrected Ca level				4.421	1.529-12.784	.006
< 10.7 mg/dl	171	6.47	.006			
≥ 10.7 mg/dl	6	0.57				
Lactate dehydrogenase						
< 250 U/L	76	10.67	.001			
≥ 250 U/L	102	3.67				
Na						
< 135 mmol/l	55	2.07	< .001			
≥ 135 mmol/l	124	9.0				
Cholesterol						
< 150 mg/dl	56	4.07	.014			
≥ 150 mg/dl	110	9.2				
Alkaline phosphatase						
< 100 U/L	80	7.03	.569			
≥ 100 U/L	99	5.63				
WBC count (× 10^3 ^cells/μL)						
< 10.0	124	10.27	< .001			
> 10.0	55	3.23				
Lymphocyte count						
< 700/μL	17	2.67	.003			
≥ 700/μL	162	7.03				
Hemoglobin level						
< 11 g/dl	67	5.33	.091			
≥ 11 g/dl	112	7.73				
Platelet count						
< 150 × 10^9^/L	33	4.87	.82			
≥ 150 × 10^9^/L	146	6.43				

HR, hazard ratio; CI, confidence interval; WBC, white blood cell.

**Figure 3:**
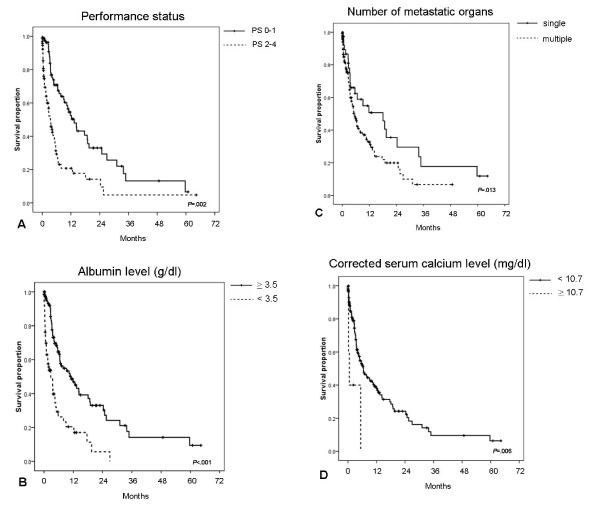
**Significant prognostic factors to overall survival in multivariate analysis**. Kaplan-Meier survival curves for patients with unfavorable CUP with (A) ECOG performance status of 0-1 vs. 2-4 (13.37 vs. 3.47 months; *P *= .002). (B) Albumin ≥ 3.5 g/dl vs. < 3.5 g/dl (11.1 vs. 2.77 months; *P *< .001). (C) Metastasis to single organ vs. multiple organs (18.07 vs. 5.33 months; *P *= .013). (D) Corrected calcium level ≥ 10.7 mg/dl vs. < 10.7 mg/dl (0.57 vs. 6.47 months; *P *= .006).

The multivariate analysis of the prognostic factors identified in the univariate analysis showed ECOG performance status ≥ 2 (hazard ratio [HR], 2.03), albumin < 3.5 g/dl (HR, 2.22), corrected calcium ≥ 10.7 mg/dl (HR, 4.42), multiple (≥ 2) metastatic sites (HR, 1.93) (Table 2) were statistically significant independent prognostic factors for shorter survival (Figure 3).

### Favorable factors for palliative chemotherapy

**Figure 4:**
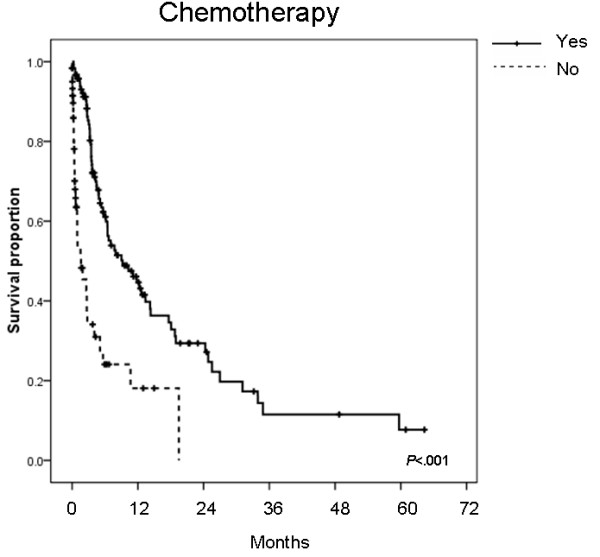
**Chemotherapy and prognosis**. Kaplan-Meier survival curves of patients with unfavorable CUP who received chemotherapy or not: yes vs. no (9.2 vs. 1.63 months; *P *= .001).

Among the patients given palliative chemotherapy, cisplatin-based regimens accounted for 75.4% of first-line chemotherapy, 43.2% regimens included 5-FU, and 11.0% were taxane-based regimens. The median overall survival of the chemotherapy group (n = 118) was 9.2 months, significantly better than the group who did not receive chemotherapy (n = 61, median survival 1.63 months, *P *< .001) (Figure 4). The differences between the two groups are shown in Table 3. The patients who received palliative chemotherapy were significantly younger (mean age, 65.5 vs. 74.4 years, *P *< .001) than patients who did not receive chemotherapy and had better performance status (*P *< .001), higher serum albumin levels (*P *= .002), and lower LDH levels (*P *= .01). Histological type or grade, number of metastatic sites, and corrected serum calcium level did not differ between the two groups.

**Table 3:** Characteristic differences between groups with/without chemotherapy

Variables	Chemotherapy		*P*
		
	Yes (n = 118)	No (n = 61)	
Median age ± SD (years)	65.62 ± 14.95	74.39 ± 10.77	< .001
Gender			.603
Male	86	42	
Female	32	19	
Albumin (g/dl)	3.71 ± 0.56	3.41 ± 0.66	.002
Corrected Ca level (mg/dl)	9.24 ± 0.57	9.35 ± 1.09	.388
Lactate dehydrogenase (U/L)	345.1 ± 300.2	574.4 ± 863.3	.01
Performance status			< .001
0	14	5	
1	57	10	
2	33	11	
3	10	17	
4	4	18	
Histology			.390
Carcinoma, unclassifiable	50	33	
Adenocarcinoma	48	23	
Squamous cell carcinoma	4	2	
Neuroendocarcinoma	10	2	
Others	6	1	
Histology grade			.151
Poorly differentiated or undifferentiated	24	7	
Others	94	54	
No. of metastatic organs			1.00
1	26	14	
≥ 2	92	47	

## Discussion

The median overall survival of CUP patients was only 3-4 months before the 1990s.^2,3 ^Recent studies identified several independent prognostic variables to predict the outcomes of patients with unfavorable CUP. Culine et al used performance status and serum LDH levels to build a prognostic index. The survival significantly differed between good and poor prognostic groups (median survival, 11.7 vs. 3.9 months, *P *< .0001) [7]. Seve et al used albumin level and liver metastasis to separate patients with unfavorable CUP into two subgroups. The low-risk group had median survival of 371 days compared to the poor-risk group that had median survival of 103 days (*P *< .0001) [9]. In the current study, in addition to the factors such as serum albumin level, performance status and number of metastatic sites, which are in consensus with previous studies [7-17], we identified two new independent prognostic factors among patients with unfavorable CUP, corrected serum calcium level (≥ 10.7 mg/dl), an indicator of poor outcome, and palliative chemotherapy, an indicator of better outcome. There are plenty of etiologies to induce hypercalcemia in cancer patients, such as osteoclastic metastases to bone, secretion of parathyroid hormone (PTH)- related peptides by tumor cells, or excessive vitamin D produced by tumors. Dismal prognoses related to hypercalcemia are observed in solid tumors and hematological malignancies [18-20]. Thus, hypercalcemia in cancer patients could universally indicate advanced illness, no matter what the primary site is.

Despite the ever-growing advances of chemotherapeutic agents in the last decade, the management of patients with unfavorable CUP remains a challenge for clinicians [21]. Recently, Kodaira et al analyzed 58 CUP patients treated with unified chemotherapy containing carboplatin and paclitaxel [11]. The overall response rate was as high as 34.5% and median overall survival was 16.7 months. Although their study did not exclude women with peritoneal carcinomatosis or adenocarcinoma, which belongs in the favorable CUP group, the high proportion of men in the sample (n = 28) and patients without adenocarcinoma (n = 32) could still imply a benefit for aggressive chemotherapy. In another cancer registry-based study done by Seve et al, overall survival was not associated significantly with chemotherapy, but marked improvement of survival in patients at cancer centers compared to those who were not could also imply a potential benefit for aggressive surveillance and treatment [8]. In the study by Culine et al, the role of chemotherapy was not analyzed even though the authors pursued the need to design a prospective trial to prove the survival benefit of palliative chemotherapy [7]. On the other hand, the meta-analysis done by Golfinopoulos et al reviewing multiple treatment regimens for CUP indicated that there was no single type of chemotherapy solidly proven to prolong the survival of patients with unfavorable CUP, although using platinum and taxane-based regimens showed possible trends of survival benefit. One limitation of this meta-analysis is that of the 10 enrolled trials published from 1980 to 2009, only four trials excluded at least some patients subsets with good prognoses [22]. In the current study, the possible survival benefit of palliative chemotherapy for certain patients with unfavorable CUP was observed and, thus, warrants further prospective studies. As shown in Table 3, it is interesting to find that patients receiving chemotherapy were significantly younger, had higher serum albumin, lower serum LDH levels, and better ECOG performance status. The above characteristics reflected the consideration of physicians for whether or not palliative chemotherapy rather than hospice care would benefit the patients with unfavorable CUP. It is not surprising that patients given chemotherapy were significantly younger than those who were not. When patients with unfavorable CUP presented with hypoalbuminemia, it was an indication of poor nutritional status, cachexia, and ongoing weight loss, and thus, discouraged the physician to administer chemotherapy [23-25]. LDH is commonly elevated in patients with actively proliferating tumor masses and is related to the tumor burden itself [26]. In the current study, poorer performance status was also associated with higher serum LDH levels (chi squared, p = .015) and was an indication not to administer chemotherapy. However, the histology subtypes were not independent prognostic factors in unfavorable subgroup of CUPs. (Table 2) Also, there were no significant differences in the distribution of variable histology groups between with and without chemotherapy treatment groups. It means that the histology type might not influence the judgement of clinician to give chemotherapy or not, and also did not influence survival.

The limitations of our study include its retrospective cohort design, the elderly median age (73 years) of the cohort, which was older than in other articles (60 years) [3], and male predominance (71.5%). This could be attributed to the patients' types found in a veterans' hospital. Besides, neuroendocrine carcinoma of unknown primary (NCUP) were not excluded in the unfavorable CUP group during data collection. Despite it is categorized into favorable CUP in the literatures, but NCUP also constituted of a heterogeneous cohort with variable histology, anatomic sites, grades, and differentiation in published series. In the latest literature review of NCUP, the median overall survival is 15.5 months [27]. In our database, the median overall survival of NCUP in the chemotherapy group is 11.1 months, and in the meanwhile, the median overall survival of all unfavorable CUP in chemotherapy group is 9.2 months. In our study, the prognosis of NCUP treated with palliative chemotherapy was not significantly better than other CUPs. This may account for why we did not exclude this group of patients in unfavorable CUP. Finally, the chemotherapy regimens were not unified, so it is difficult to compare the efficacy between specific drugs. However, the variety of treatment regimens could also reflect the lack of standard treatment guidelines for treatment of unfavorable CUP at present [22].

## Conclusion

In conclusion, our study identified several independent prognostic factors in patients with unfavorable CUP. It also demonstrated that better performance status, higher serum albumin levels, and lower LDH levels may influence the decision of the clinican to give palliative chemotherapy, which might be associated with longer survival in unfavorable CUP patients. This study provide further characterization of patients with unfavorable CUP. Whether selection of individuals who would benefit from chemotherapy warrants prospective studies in the future.

## List of abbreviations used

CUP: Carcinoma of unknown primary site; ECOG: Eastern Cooperative Oncology Group; HR: hazard ratio; IRB: institutional review board; LDH: lactate dehydrogenase; NCUP: neuroendocrine carcinoma of unknown primary; PTH: parathyroid hormone.

## Competing interests

The authors declare that they have no competing interests.

## Authors' contributions

K-WC carried out the data collection, analysis of the data and manuscript preparation. C-JL had participated in the data collection and statistical assistance and also the manuscript writing. H-JL had joined in the data analysis and manuscript writing. C-HT, J-HL, T-JC, C-CY, W-SW, T-CC, H-WT, M-HC, C-YL have contributed to the study design, acquisition of the data. PM-HC is the correspondence of this study, participated in study design, critical appraisal of the manuscript and revision of the writing. M-H Yang is the mentor of PM-HC and also had participated in data acquisition. All authors read and approved the final manuscript.

## References

[B1] Greco FA Hainsworth JD DeVita Jr VT, Lawrence TS, Rosenberg SA Cancer of unknown primary site DeVita, Hellman, Rosenberg's Cancer: Principles and Practice of Oncology 2001 6 Philadelphia: Lippincott Williams & Wilkins 2537 2560

[B2] Krementz ET Cerise EJ Foster DS Morgan LR Jr Metastases of undetermined source Curr Probl Cancer 1979 4 4 37 391494 10.1016/s0147-0272(79)80019-7

[B3] Pavlidis N Briasoulis E Hainsworth J Greco FA Diagnostic and therapeutic management of cancer of an unknown primary Eur J Cancer 2003 39 1990 2005 10.1016/S0959-8049(03)00547-1 12957453

[B4] Muir C Cancer of unknown primary site Cancer 1995 75 1 Suppl 353 356 8001006 10.1002/1097-0142(19950101)75:1+<353::aid-cncr2820751317>3.0.co;2-p

[B5] Greco FA Pavlidis N Treatment for patients with unknown primary carcinoma and unfavorable prognostic factors Semin Oncol 2009 36 65 74 10.1053/j.seminoncol.2008.10.005 19179190

[B6] Varadhachary GR Greco FA Overview of patient management and future directions in unknown primary carcinoma Semin Oncol 2009 36 75 80 10.1053/j.seminoncol.2008.10.008 19179191

[B7] Culine S Kramar A Saghatchian M Development and validation of a prognostic model to predict the length of survival in patients with carcinomas of an unknown primary site J Clin Oncol 2002 20 4679 4683 10.1200/JCO.2002.04.019 12488413

[B8] Seve P Sawyer M Hanson J Broussolle C Dumontet C Mackey JR The influence of comorbidities, age, and performance status on the prognosis and treatment of patients with metastatic carcinomas of unknown primary site: a population-based study Cancer 2006 106 2058 2066 10.1002/cncr.21833 16583433

[B9] Seve P Ray-Coquard I Trillet-Lenoir V Low serum albumin levels and liver metastasis are powerful prognostic markers for survival in patients with carcinomas of unknown primary site Cancer 2006 107 2698 2705 10.1002/cncr.22300 17063500

[B10] Trivanović D Petkovic M Stimac D New prognostic index to predict survival in patients with cancer of unknown primary site with unfavourable prognosis Clin Oncol 2009 21 43 48 10.1016/j.clon.2008.09.007 18976894

[B11] Kodaira M Takahashi S Yamada S Bone metastasis and poor performance status are prognostic factors for survival of carcinoma of unknown primary site in patients treated with systemic chemotherapy Ann Oncol 2010 21 1163 1167 10.1093/annonc/mdp583 20019088

[B12] Kambhu SA Kelsen DP Fiore J Metastatic adenocarcinomas of unknown primary site. Prognostic variables and treatment results Am J Clin Oncol 1990 13 55 60 10.1097/00000421-199002000-00015 2106257

[B13] Abbruzzese JL Abbruzzese MC Hess KR Raber MN Lenzi R Frost P Unknown primary carcinoma: natural history and prognostic factors in 657 consecutive patients J Clin Oncol 1994 12 1272 1280 8201389 10.1200/JCO.1994.12.6.1272

[B14] van der Gaast A Verweij J Planting AS Hop WC Stoter G Simple prognostic model to predict survival in patients with undifferentiated carcinoma of unknown primary site J Clin Oncol 1995 13 1720 1725 7541451 10.1200/JCO.1995.13.7.1720

[B15] Viagano A Bruera E Jhangri GS Newman SC Fields AL Suarez-Almazor ME Clinical survival predictors in patients with advanced cancer Arch Intern Med 2000 160 861 868 10.1001/archinte.160.6.861 10737287

[B16] Dixon MR Haukoos JS Udani SM Carcinoembryonic antigen and albumin predict survival in patients with advanced colon and rectal cancer Arch Surg 2003 138 962 966 10.1001/archsurg.138.9.962 12963652

[B17] Parker D Bradley C Bogle SM Serum albumin and CA 125 are powerful predictors of survival in epithelial ovarian cancer Br J Obstet Gynaecol 1994 101 888 893 10.1111/j.1471-0528.1994.tb13550.x 7999691

[B18] Alexanian R Balcerzak S Bonnet JD Prognostic factors in multiple myeloma Cancer 1975 36 1192 201 10.1002/1097-0142(197510)36:4<1192::AID-CNCR2820360403>3.0.CO;2-I 1175123

[B19] Won C Decker DA Drelichman A Al-Sarraf M Reed ML Hypercalcemia in head and neck carcinoma. Incidence and prognosis Cancer 1983 52 2261 2263 10.1002/1097-0142(19831215)52:12<2261::AID-CNCR2820521217>3.0.CO;2-K 6640497

[B20] De Wit S Cleton FJ Hypercalcemia in patients with breast cancer: a survival study J Cancer Res Clin Oncol 1994 120 610 614 10.1007/BF01212816 7929533 PMC12201004

[B21] Hainsworth JD Greco FA Treatment of patients with cancer of an unknown primary site N Engl J Med 1993 329 257 263 10.1056/NEJM199307223290407 8316270

[B22] Golfinopoulos V Pentheroudakis G Salanti G Nearchou AD Ioannidis JP Pavlidis N Comparative survival with diverse chemotherapy regimens for cancer of unknown primary site: multiple-treatments meta-analysis Cancer Treat Rev 2009 35 570 573 10.1016/j.ctrv.2009.05.005 19539430

[B23] Chihara D Oki Y Ine S Analysis of prognostic factors in peripheral T-cell lymphoma: prognostic value of serum albumin and mediastinal lymphadenopathy Leuk Lymphoma 2009 50 1999 2004 10.3109/10428190903318311 19860627

[B24] Al-Shaiba R McMillan DC Angerson WJ Leen E McArdle CS Horgan P The relationship between hypoalbuminaemia, tumour volume and the systemic inflammatory response in patients with colorectal liver metastases Br J Cancer 2004 91 205 207 15213726 10.1038/sj.bjc.6601886 PMC2409827

[B25] McMillan DC Watson WS O'Gorman P Preston T Scott HR McArdle CS Albumin concentrations are primarily determined by the body cell mass and the systemic inflammatory response in cancer patients with weight loss Nutr Cancer 2001 39 210 213 10.1207/S15327914nc392_8 11759282

[B26] Schneider RJ Seibert K Passe S Prognostic significance of serum lactate dehydrogenase in malignant lymphoma Cancer 1980 46 139 143 10.1002/1097-0142(19800701)46:1<139::AID-CNCR2820460122>3.0.CO;2-8 6992974

[B27] Stoyianni A Pentheroudakis G Pavlidis N Neuroendocrine carcinoma of unknown primary: a systematic review of the literature and a comparative study with other neuroendocrine tumors Cancer Treat Rev 2011 37 358 65 10.1016/j.ctrv.2011.03.002 21481536

